# Intraneuronal APP and extracellular Aβ independently cause dendritic spine pathology in transgenic mouse models of Alzheimer’s disease

**DOI:** 10.1007/s00401-015-1421-4

**Published:** 2015-04-11

**Authors:** Chengyu Zou, Elena Montagna, Yuan Shi, Finn Peters, Lidia Blazquez-Llorca, Song Shi, Severin Filser, Mario M. Dorostkar, Jochen Herms

**Affiliations:** Department for Translational Brain Research, German Center for Neurodegeneratione Diseases (DZNE), Ludwig-Maximilians-University Munich, Munich, Germany; Center for Neuropathology and Prion Research, Ludwig-Maximilians-University Munich, Feodor-Lynen-Straße 23, 81377 Munich, Germany; Munich Cluster of Systems Neurology (SyNergy), Ludwig-Maximilians-University Munich, Schillerstraße 44, 80336 Munich, Germany; Graduate School of Systemic Neuroscience, Ludwig-Maximilians-University Munich, Munich, Germany

**Keywords:** Alzheimer’s disease, Intraneuronal APP, Extracellular Aβ, Dendritic spines, Two-photon in vivo imaging

## Abstract

Alzheimer’s disease (AD) is thought to be caused by accumulation of amyloid-β protein (Aβ), which is a cleavage product of amyloid precursor protein (APP). Transgenic mice overexpressing APP have been used to recapitulate amyloid-β pathology. Among them, APP23 and APPswe/PS1deltaE9 (deltaE9) mice are extensively studied. APP23 mice express APP with Swedish mutation and develop amyloid plaques late in their life, while cognitive deficits are observed in young age. In contrast, deltaE9 mice with mutant APP and mutant presenilin-1 develop amyloid plaques early but show typical cognitive deficits in old age. To unveil the reasons for different progressions of cognitive decline in these commonly used mouse models, we analyzed the number and turnover of dendritic spines as important structural correlates for learning and memory. Chronic in vivo two-photon imaging in apical tufts of layer V pyramidal neurons revealed a decreased spine density in 4–5-month-old APP23 mice. In age-matched deltaE9 mice, in contrast, spine loss was only observed on cortical dendrites that were in close proximity to amyloid plaques. In both cases, the reduced spine density was caused by decreased spine formation. Interestingly, the patterns of alterations in spine morphology differed between these two transgenic mouse models. Moreover, in APP23 mice, APP was found to accumulate intracellularly and its content was inversely correlated with the absolute spine density and the relative number of mushroom spines. Collectively, our results suggest that different pathological mechanisms, namely an intracellular accumulation of APP or extracellular amyloid plaques, may lead to spine abnormalities in young adult APP23 and deltaE9 mice, respectively. These distinct features, which may represent very different mechanisms of synaptic failure in AD, have to be taken into consideration when translating results from animal studies to the human disease.

**Electronic supplementary material:**

The online version of this article (doi:10.1007/s00401-015-1421-4) contains supplementary material, which is available to authorized users.

## Introduction

Alzheimer’s disease (AD) is the most prevalent cause of dementia and currently no effective treatment exists. Multiple strands of evidence suggest that amyloid precursor protein (APP) and its proteolytic fragment, amyloid β-protein (Aβ), play a crucial role in the pathogenesis of AD [62]. APP is a single-pass transmembrane protein enriched at synapses [19]. The highly conserved APP gene is located on chromosome 21 and overexpression of APP in Down’s syndrome (trisomy 21) causes accumulation of amyloid plaques early in life [21]. Through sequential enzymatic cleavage by β and γ-secretases, full-length APP is processed to yield amyloid beta (Aβ) as well as other fragments. Accumulation of fibrillar Aβ leads to formation of senile plaques, the typical neuropathological hallmark of AD. Soluble oligomeric Aβ, in contrast, is thought to mediate synapse dysfunction and loss, which strongly correlate with cognitive decline in AD [20, 32]. The amyloid hypothesis takes the imbalance between Aβ production and clearance as the primary cause of AD [20]. Based on this hypothesis and the discovery of familial AD mutations that facilitate Aβ production, transgenic mouse models overexpressing mutant APP and/or presenilins (PS), which form part of the γ-secretase complex, have been created to recapitulate AD pathology.

Among the APP transgenic mouse models, APP23 and APPswe/PS1deltaE9 (deltaE9) mice have been extensively used for exploring AD-related pathology and drug development [63]. To recapitulate the pathogenesis of human AD, APP23 mouse model overexpresses human APP with the Swedish mutation under the murine Thy1 promoter [57], while deltaE9 mice express APP with the Swedish mutation controlled by mouse prion protein promoter elements together with mutant human PS1 lacking exon 9, which is associated with familial AD [29, 52]. Although these two transgenic mouse models display neuronal loss, cholinergic deficit, cognitive impairments, amyloid plaques and neuroinflammation in old age, the onsets of amyloid plaque formation and cognitive decline between them are very different in early adulthood [5, 8, 9, 30, 38, 56]. Aβ deposits are not observed in APP23 mice younger than 6 months, but age-matched deltaE9 mice have already developed plaques [28]. Despite the slower progress of amyloid plaque formation, APP23 mice show faster cognitive decline than deltaE9 mice. APP23 mice begin to develop cognitive deficits at 3 months, while deltaE9 mice do not have typical impaired memory until 1 year of age [60, 61]. Uncovering and understanding the discrepancies between them are important for the utility of particular animal models to deepen our knowledge of synaptic failure in AD.

Using in vivo two-photon imaging of cortical layer V pyramidal neurons, we found reduced dendritic spine density in 4–5-month-old APP23 mice. In age-matched deltaE9 mice, loss of dendritic spines was only observed in close proximity to plaques. Furthermore, chronic in vivo imaging revealed that spine loss in AD transgenic mouse models was the consequence of decreased spine formation. Also, morphologies of dendritic spines in APP23 and deltaE9 mice were altered differently. Immunostaining showed accumulated intracellular APP in APP23 mice. The amount of intracellular APP was negatively correlated with spine density and morphology. These results suggest that spine abnormalities in young adult APP23 and deltaE9 mice might be caused by intracellular APP and extracellular Aβ deposits, respectively.

## Materials and methods

### Animals

APP23 (Novartis) and APPswe/PS1deltaE9 mice (Jackson Laboratory) [29, 52, 57] were crossed with GPF-M mice (Jackson Laboratory) [13] to obtain double-transgenic offspring heterozygous for the corresponding genes. All transgenic lines were kept on C57BL/6 background. eGFP-positive littermates without mutant APP and PS1 transgenes were used as controls. Only female mice at the age of 4–5 months were used in this study. All protocols and procedures were conducted according to the animal protocol approved by the Ludwig-Maximilian University Munich and the government of Upper Bavaria.

### Cranial window implantation and in vivo two-photon imaging

As previously described [24], mice were anesthetized by intraperitoneal injection of ketamine/xylazine (130/10 μg/g body weight). Subsequently, dexamethasone (6 μg/g body weight) was injected intraperitoneally to prevent development of cerebral edema. A piece of skull above the somatosensory cortex was removed and replaced with a 4-mm-diameter coverslip. After a 4-week recovery period, apical dendrites originating from layer V pyramidal neurons were imaged using a LSM 7MP microscope (Zeiss) equipped with a 20× water-immersion objective (1.0 NA, Zeiss). Mice were anesthetized with isoflurane and placed on a heating pad to maintain the body temperature. Any single imaging session lasted no longer than 1 h. In subsequent imaging sessions, imaged regions were re-localized based on the unique pattern of blood vessels. To stain amyloid plaques in vivo, methoxy-X04 (1 mg/kg) was intraperitoneally injected 24 h before imaging. For overview images, 424 × 424 × 350 µm^3^ z-stacks (0.83 µm/pixel) were taken. Higher resolution images (0.138 µm/pixel) were used for counting dendritic spines.

### Spine analysis

Spines were counted manually in ZEN 2011 (Zeiss). Due to limitations in resolution in the Z-direction, only laterally protruding spines were taken into account, as only those could be identified with certainty and classified morphologically. Spines that had emerged or disappeared since the previous imaging session were classified as formed or eliminated, respectively. Spine turnover rate (TOR) was calculated as follows: (*N*_f_ + *N*_e_)/(2 × *N*_t_ × *D*), *N*_f_ = formed spines, *N*_e_ = eliminated spines, *N*_t_ = total spines, *D* = interval days between imaging sessions. For morphological analysis, maximum intensity projections from in vivo two-photon stacks were used. The length of each spine was measured from the tip of the spine head to the bottom of the spine neck. Spine head width was defined as the length between the left edge of spine head and the right edge. Spines were classified into mushroom, stubby and thin spines based on their appearances as described before [22, 24].

### Immunohistochemistry

Following transcardial perfusion with phosphate-buffered saline (PBS) and 4 % paraformaldehyde (PFA), mouse brains were fixed in 4 % PFA overnight at 4 °C and then cut into 65-µm-thick free-floating frontal sections at the level of the somatosensory cortex. β amyloid (4G8, Covance, 1:200), beta-amyloid 40 (139-5, Covance, 1:100), and beta-amyloid 42 (11-1-3, Covance, 1:100) and anti-APP 22C11 (Millipore, 1:20) antibodies were used for APP and Aβ staining. Anti-mouse or rabbit Alexa 647 antibody (Life technologies, 1:1000) was used as the secondary antibody. For spine imaging, sections were incubated with anti-GFP coupled with Alexa 488 (Life technologies, 1:300) and then mounted on glass coverslips using fluorescence mounting medium (Dako). For the microscopy of cortical areas, LSM 780 confocal microscope (Zeiss) was equipped with a 10×/0.3 objective. To image pyramidal neurons and dendrites, a 40×/1.4 objective was used and 212 × 212 × 80 µm^3^ z-stacks (0.415 µm/pixel) were taken for overview images and APP quantification. To quantify the relative APP amount, custom-written Matlab software was applied to correct for the depth-dependent changes inherent to data obtained from brain slices immunostained with fluorophor-coupled antibodies. Exponential fitting was applied to correct for the reduction in fluorescence intensity toward the center of the brain slice due to decreasing antibody penetration as well as the additional reduction imposed by light scattering and light absorption over the complete depth of the slice. Higher resolution images (0.069 µm/pixel) were used for counting dendritic spines.

### Statistics

Analyses were performed blinded with respect to mouse genotype. The numbers of mice for in vivo two-photon imaging were 5–6 per group. 7–12 dendrites were imaged in each mouse; the length of each dendrite was 25–35 µm. The data are presented as the means for every mouse (round symbols) and the means of the means (horizontal line with error bars), except for the data shown in Fig. 3, where the data from 13 dendrites out of 5 mice, which were located in proximity to nascent plaques (50–80 µm), are shown. More than 30 neurons from 5 mice were imaged in ex vivo imaging. Results are presented as mean ± SEM and compared with controls by one-way ANOVA with Dunnett’s test. Kolmogorov–Smirnov test was used for comparing cumulative frequency distributions. Extra sum-of-squares *F* test was used when data were fitted a straight line with nonlinear regression. *p* < 0.05 was defined as statistically significant with *p < 0.05, **p < 0.01, N.S.: not significant.

## Results

### Dendritic spine density of layer V pyramidal neurons is reduced differently in young adult APP23 and deltaE9 mice

In this study, we used APP23 and deltaE9 mouse models, which both express human APP with the Swedish mutation. In deltaE9 mice, mutant human PS1 lacking exon 9 is co-expressed [29, 52, 57]. These two mouse models develop neuropathological hallmarks of AD differently in young adulthood. APP23 mice show cognitive deficits before amyloid plaque formation while deltaE9 mice develop memory loss after Aβ deposition [28, 60, 61].

**Fig. 1:**
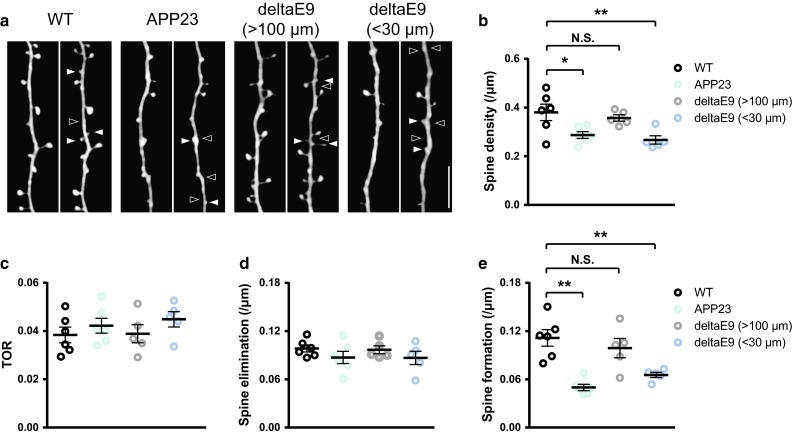
Decreased spine density in dendrites of APP23 mice and deltaE9 mice. **a** By in vivo two-photon imaging, the same apical dendrites from layer V pyramidal neurons in the somatosensory cortex were repeatedly imaged 1 week apart. Each image is a maximum intensity projection of serial sections. *White arrowheads* point at spines formed over 1 week and *empty arrowheads* point at eliminated spines. *Scale bar* 10 μm. **b** Spine densities of apical dendrites in WT, APP23 and deltaE9 mice. In deltaE9 mice, dendrites that were localized at plaque-free overview images are classified as deltaE9 (>100 µm) and the ones in close proximity to plaques are named as deltaE9 (<30 µm). **c** Turnover rates of apical dendrites in WT, APP23 and deltaE9 (>100 µm and <30 µm) mice. **d**, **e** Spines that were eliminated (**d**) and newly formed (**e**) over 1 week in WT, APP23 and deltE9 (>100 µm and <30 µm) mice. In WT group, *n* = 6. In APP23 group, *n* = 6. In deltaE9 (>100 µm) group, *n* = 5. In deltaE9 (<30 µm) group, *n* = 5. **p < 0.01 (ANOVA with Dunnett’s post hoc test)

To examine whether and how AD transgenic mouse models develop synaptic pathology in young adulthood, we crossed APP23 and deltaE9 mice with GFP-M transgenic mice to visualize apical dendrites of layer V pyramidal neurons by in vivo two-photon microscopy (Fig. 1a). We found a significant decrease of spine density in APP23 mice at the age of 4–5 months (0.28 ± 0.01 spines/µm, vs. WT 0.38 ± 0.03 spines/µm, Fig. 1b). Because Aβ deposits emerge in deltaE9 mice as early as 4 months of age and amyloid plaques disturb dendritic spine stability [3, 16], we analyzed dendrites in deltaE9 mice that were close and far from plaques. Dendrites that were chosen from plaque-free overview images (>100 µm from plaques, supplementary Figure 1a) did not show spine loss (0.36 ± 0.01 spines/µm, Fig. 1b), but the ones that were in close proximity to plaques (<30 µm from plaques, supplementary Figure 1b) displayed a strong decrease in spine density (0.27 ± 0.02 spines/µm, Fig. 1b).

### Imbalance between spine formation and elimination causes spine loss

To determine whether spine dynamics are altered in APP23 and deltaE9 mice, we repeatedly imaged apical dendrites 1 week apart in the somatosensory cortex. While the spine turnover rate in both AD models did not differ from WT animals (0.038 ± 0.003 vs. 0.042 ± 0.003 vs. 0.039 ± 0.004 vs. 0.045 ± 0.003, Fig. 1c), we found that significantly fewer new spines emerged in APP23 mice (0.05 ± 0.004 spines/µm, vs. WT 0.11 ± 0.01 spines/µm, Fig. 1e). In deltaE9 mice, spine formation was also decreased on dendrites that were in proximity to plaques (<30 µm, 0.065 ± 0.003 spines/µm, Fig. 1e), but not on dendrites far away from plaques (>100 µm, 0.099 ± 0.01 spines/µm, Fig. 1e). The spine eliminations among WT, APP23 and deltaE9 mice (>100 µm and <30 µm) were comparable (0.098 ± 0.004 spines/µm vs. 0.087 ± 0.008 spines/µm vs. 0.097 ± 0.005 spines/µm vs. 0.087 ± 0.008 spines/µm, Fig. 1d). These results suggest that the decrease in the spine density of young adult AD mice is a consequence of an imbalance between spine formation and elimination.

### Alterations in spine morphology differ between APP23 and deltaE9 mice

**Fig. 2:**
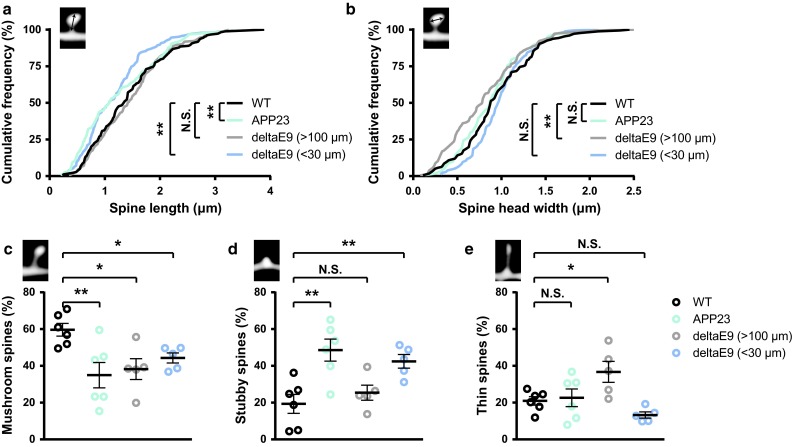
Dendritic spine morphology changes differently in APP23 and deltaE9 mice. **a**, **b** Cumulative distributions of spine length (**a**) and spine head width (**b**) in WT, APP23 and deltaE9 (>100 µm and <30 µm) mice. **a**, **b** **p < 0.01 (Komogornov–Smirnov test). **c**–**e** Fractions of mushroom (**c**), stubby (**d**) and thin spines (**e**). Representative classified spines are on the *top-left corner*. In WT group, *n* = 6. In APP23 group, *n* = 6. In deltE9 (>100 µm) group, *n* = 5. In deltE9 (<30 µm) group, *n* = 5. **c**–**e** *p < 0.05, **p < 0.01 (ANOVA with Dunnett’s post hoc test)

Besides absolute spine density, spine morphology also correlates with dendritic spine function and thus impacts cognitive performance [51]. To examine whether the spine morphology of these AD transgenic mouse models is altered, we measured spine length and spine head width of the in vivo imaged dendritic spines. Spine lengths of dendritic spines from APP23 and deltaE9 mice (<30 µm) were significantly decreased, while spines from deltaE9 mice (>100 µm) showed decreased spine head width (Fig. 2a, b). Moreover, we classified the spines according to their morphological appearance into mushroom, stubby and thin spines [24]. APP23 and deltaE9 mice (>100 µm and <30 µm) showed a reduced fraction of mushroom spines (35.0 ± 6.9 %, 38.2 ± 5.7 % and 44.3 ± 2.7 % vs. WT 59.6 ± 3.5 %, Fig. 2c). Furthermore, in APP23 mice and deltaE9 mice (<30 µm), the decreases of mushroom spines were accompanied with strong increases in the stubby spines (48.6 ± 6.0 % and 42.5 ± 3.7 % vs. WT 19.4 ± 5.2 %, Fig. 2d). However, thin spines, but not stubby spines, were increased in deltaE9 mice (>100 µm, 36.7 ± 5.7 % vs. WT 20.9 ± 2.3 %, Fig. 2e). Collectively, these results show that morphological alterations of dendritic spines in APP23 and deltaE9 mice, are distinct. In addition, these alterations even differ between different distances to fibrillar plaques within deltaE9 mice.

### Spine loss and alterations in spine morphology are associated with amyloid plaque growth in deltaE9 mice

**Fig. 3:**
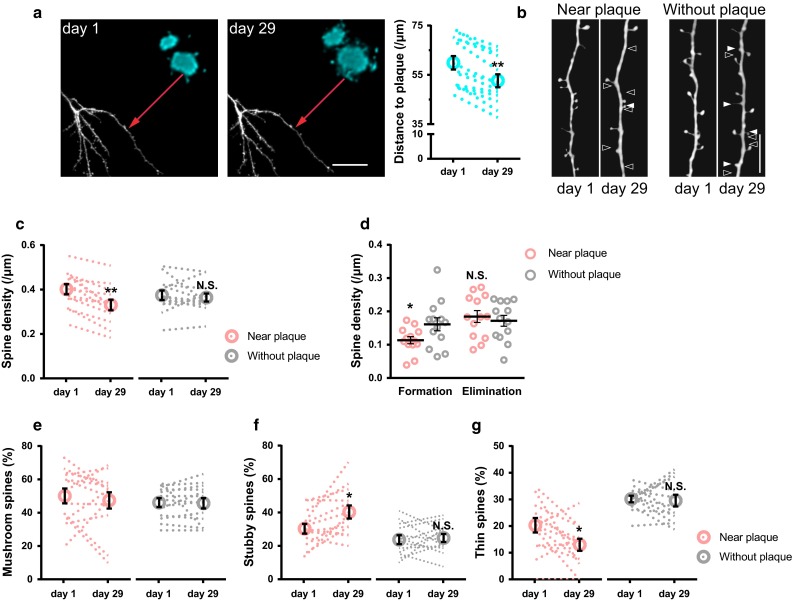
Spine loss and morphological alterations are accompanied by amyloid plaque growth in deltaE9 mice. **a** Maximum intensity projections of two-photon in vivo images of GFP-labeled dendrites (*white*) and methoxy-X04-labeled plaques (*blue*) are shown. The distance from dendrite to plaque (*red arrow line*) is reduced after 1 month due to plaque growth. *Scale bar* 40 μm. **b** Maximum intensity projected dendrites from a (*arrowhead* pointed, near plaque) and from plaque-free overview images (without plaque) in deltaE9 mice. *Scale bar* 10 μm. **c** Spine densities of the dendrites that were near plaque or in plaque-free area over 1 month. Each *dashed line* represents one dendrite. **d** Newly formed and eliminated spine densities of the dendrites that were near plaque or in plaque-free area over 1 month. **e**–**g** Fractions of mushroom (**e**), stubby (**f**) and thin spines (**g**) in these two different dendrites over 1 month. Each *dashed line* represents one dendrite. Paired *t* test was used for plaque growth-mediated spine alterations and unpaired *t* test was used to compare spine formation and elimination between groups. *n* = 13 in each group. *p < 0.05, **p < 0.01

In young adult deltaE9 mice, dendrites that were located near (<30 µm) and far (>100 µm) away from plaques displayed two different patterns of spine abnormalities. Close to plaques (<30 µm) a decrease in spine density and increase in the fraction of stubby spines were observed. Dendrites far away from plaques (>100 µm) did not develop spine loss but showed increased fraction of thin spines. To investigate whether the alterations of dendritic spine abnormalities are correlated with the distance between dendrites and plaques, we imaged dendrites that resided 50–80 µm away from plaques. With amyloid plaque growth over 1 month, the distance between dendrites and plaques became smaller (from 59.9 ± 2.7 µm to 52.6 ± 2.6 µm, Fig. 3a). Meanwhile, dendrites started to develop spine loss (Fig. 3b, c). The decrease of spine density was caused by reduced spine formation (Fig. 3d). Moreover, the fraction of mushroom spines remained unchanged (Fig. 3e), while the fraction of stubby spines increased along with the decrease of thin spine fraction (Fig. 3f, g). Taken together, these results indicate that amyloid plaques cause manifold dendritic spine alterations in deltaE9 mice.

### APP accumulates intracellularly in APP23 mice

**Fig. 4:**
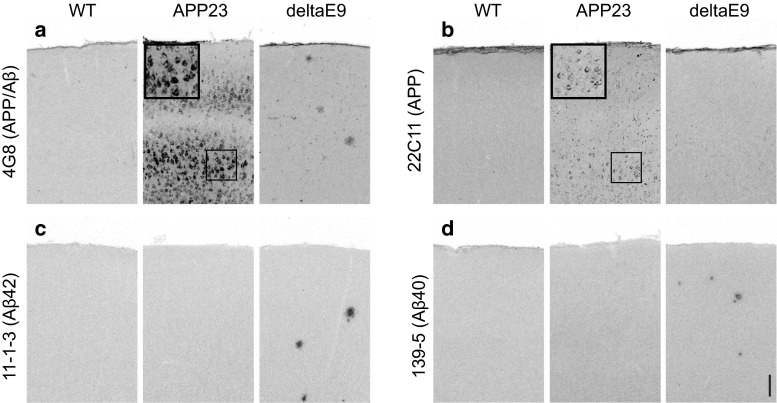
Intracellular accumulation of APP in APP23 mice. **a**–**d** Immunohistochemical labeling of intracellular APP/Aβ (4G8, **a**), intracellular APP (22c11, **b**), Aβ42 deposits (11-1-3, **c**) and Aβ40 deposits (139-5, **d**) in WT, APP23 and deltaE9 mice. *Scale bar* 100 μm

To exclude the possibility that the decreased spine density which we observed in APP23 mice was not caused by the close vicinity to amyloid plaques [4], we used methoxy-X04 to label fibrillar amyloid deposits in vivo [7] and no plaque was found in the imaged volumes in APP23 mice at the age of 4–5 months(data not shown). Ex vivo immunohistochemical staining with methoxy-X04 further confirmed that APP23 mice had not yet developed amyloid plaques (data not shown). Furthermore, we stained brain sections using an antibody that recognizes both APP and Aβ (4G8). Surprisingly, a strong APP/Aβ somatic staining was observed in the cortex of 4–5-month-old APP23 mice (Fig. 4a). To further clarify the identity of the intracellular immunoreactivity, we used antibodies specific to detect Aβ40, Aβ42 and APP. No intracellular immunoreactivity was detected by Aβ-specific antibodies in APP23 mice (Fig. 4c, d). The ability of these antibodies to bind Aβ peptides was verified by the detection of extracellular Aβ deposits in deltaE9 mice (Fig. 4c, d). In contrast, intracellular APP immunoreactivity was also observed with the APP-specific antibody 22C11 in APP23 mice (Fig. 4b). Western blot analysis further confirmed APP23 mice mainly overexpressed full-length APP, but not Aβ, in young adulthood (Supplementary Figure 2). Notably, the expression of APP in APP23 mice was higher than in deltaE9 mice (Supplementary Figure 2), which is in line with previous reports [28, 57]. These results suggest that the intracellular accumulations in APP23 mice consist of APP, rather than Aβ.

### The amount of intracellular APP correlates with dendritic spine alterations

**Fig. 5:**
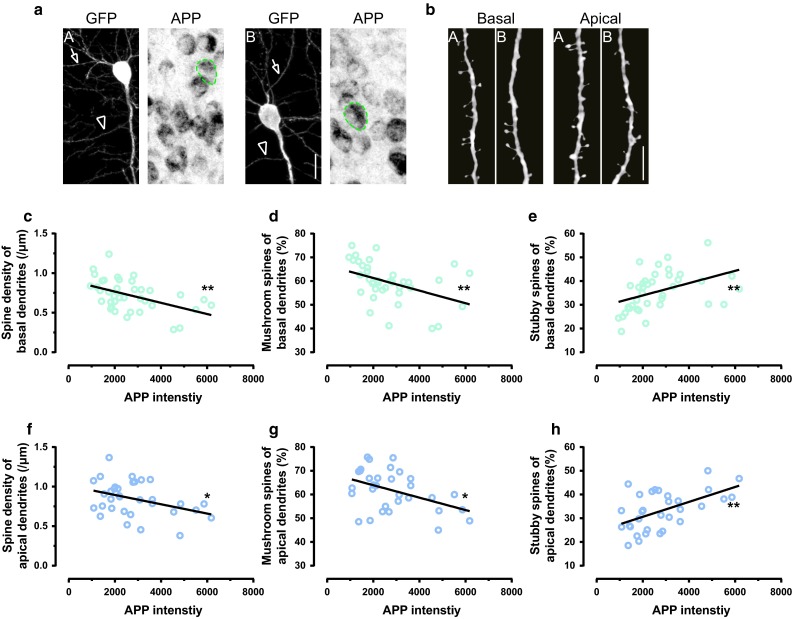
Increased intracellular APP accumulation is accompanied with decreased spine density and altered spine morphologies in the somatosensory cortex of APP23 mice. **a** Maximum intensity projections of ex vivo images of GFP-labeled neurons (*white*, *A* and *B*) and intracellular APP accumulation in layer V pyramidal neurons (*black*). *Green dashed circle* indicates the area of soma from GFP-labeled neurons. *Arrows* and *arrow heads* point to basal and apical dendrites, respectively. *Scale bar* 20 μm. **b** Maximum intensity projected basal and apical dendrites from *A* and *B*. *Scale bar* 10 μm. **c**–**e** The *dot plots* are the intensity of intracellular APP in basal dendrites from layer V pyramidal neurons vs. spine density, mushroom and stubby fractions separately. *Straight lines* are fitted by nonlinear regression. Each *dot* represents one neuron. **f**–**h** The *dot plots* are the intensity of intracellular APP in apical dendrites from layer V pyramidal neurons vs. spine density, mushroom and stubby fractions separately. *Straight lines* are fitted by nonlinear regression. Each *dot* represents one neuron. In basal dendrite group, *n* = 38. In apical dendrite group, *n* = 33 (**c**–**h**) *p < 0.05, **p < 0.01 (*F* test)

In young adult APP23 mice, spine density of cortical pyramidal neurons was reduced and spine morphology was also changed. To assess if these structural alterations were caused by the observed intracellular APP accumulation, the amount of APP in the soma of eGFP-labeled cortical layer V pyramidal neurons was quantified from brain sections (Fig. 5a). Along with the increase of intracellular APP, spine densities on apical and basal dendrites of pyramidal neurons declined (Fig. 5b, c, f). In addition, the fractions of mushroom spines were decreased (Fig. 5d, g), while stubby spine fractions were increased (Fig. 5e, h). Besides, the accumulation of intracellular APP in CA1 pyramidal neurons also coincided with the decrease of spine density and alterations of spine morphology (Supplementary Figure 3). Altogether, these results suggest that intracellular accumulation of APP may be responsible for the spine alterations in 4–5-month-old APP23 mice.

## Discussion

Extracellular Aβ is accepted to be in the center of AD pathogenesis due to its neurotoxicity that disrupts multiple physiological processes [53]. Guided by the amyloid hypothesis, AD mouse models have been created to recapitulate the cognitive impairments seen in AD patients. These mouse models typically express human APP with or without PS1 with familial AD mutations, which both cause familial forms of AD. Although most of the mouse models develop typical amyloid plaques and cognitive deficits with age, the pathophysiology in young transgenic mice, reflecting preclinical forms of AD, is less well understood [63]. APP23 mice display cognitive impairments before plaque formation, while deltaE9 mice develop abundant plaques before the decline of cognitive performance. The underlying mechanisms of these discrepancies are still not clear.

The major correlate of cognitive impairment is synapse loss, which is closely associated with spine loss as excitatory glutamatergic synapses normally reside at dendritic spines in the mammalian brain [43]. In addition to absolute spine density, the dynamic turnover of spines, termed structural plasticity, is also involved with learning and memory: the formation and elimination of dendritic spines rewire neural circuits by establishing or abolishing connections in the brain during learning experiences [15]. Thus, it is plausible to examine alterations of dendritic spines as readout for structural correlate of cognitive decline in AD transgenic mouse models.

In this study, we found that 4–5-month-old APP23 mice displayed reduced spine density of cortical layer V pyramidal neurons. In deltaE9 mice, spine loss was only evident on dendrites that were located close to plaques. We found similar results in the APPswe/PS1L166P mouse model [48], which accumulates plaques faster than the deltaE9 model: here, spines were lost only in the vicinity (<50 µm) of plaques, while spines were not altered distant (>50 µm) to plaques or before plaques had appeared [3]. These results suggest that spine loss mediated by fibrillar amyloid plaques occurs only in the immediate vicinity of extracellular Aβ deposits in deltaE9 and APPswe/PS1L166P mice.

The decreased spine densities observed in APP23 and deltaE9 mice were caused by reduced spine formation as revealed by chronic repetitive in vivo two-photon imaging. Interestingly, we found two different patterns of spine morphological alterations in these two transgenic mouse models. In APP23 mice, the spine length was reduced and the relative proportion of stubby spines was increased. In deltaE9 mice, in dendrites close to plaques, the findings were identical. In contrast, the dendrites that were far away from plaques in deltaE9 mice showed decreased spine head width and elevated thin spine fraction. With amyloid plaque growth in deltaE9 mice, dendrites, that were originally located 50–80 µm away from plaques, became closer to plaques and started to lose spines. This effect was accompanied with an increase in the fraction of stubby spines. In APP23 mice, APP accumulated intracellularly. A higher content of APP was inversely correlated with spine density. Furthermore, an increased fraction of mushroom spines and decreased fraction of stubby spines were observed in neurons, which contained higher levels of intracellular APP. In summary, our data suggested that different pathological mechanisms, intracellular APP and extracellular amyloid plaques, might lead to spine abnormalities in young adult APP23 and deltaE9 mice, respectively.

Dendritic spines are the small bulbous postsynaptic elements of the majority of excitatory synapses and serve as the basic units for learning and memory [22]. Loss of dendritic spines is the major correlate of cognitive impairment in human AD [59]. In agreement with the spine loss described before, APP23 mice younger than 6 months show memory impairments in multiple cognitive tests, including Morris-type water maze test, Y-maze test, Barnes-maze test and novel-object recognition test [12, 25, 31, 60]. On the other hand, the performance of deltaE9 mice at the same age is normal in most cognitive tests (T-maze test, Y-maze test, Morris-type water maze test, novel taste neophobia test, response acquisition test, Barnes-maze spatial memory task with hidden-target strategies), with the exception of impairments observed in Barnes-maze spatial memory task with cued-target strategies and modified radial-arm water maze test [18, 34, 45, 49, 61]. The specific spatial learning deficit described in young deltaE9 mice may depend on dendritic spine shape, rather than a reduced spine number, considering that spine loss is only observed on dendrites that are localized very close to amyloid plaques, which just start to emerge in 4–5-month-old deltaE9 mice [6, 16, 28]. With aging, Aβ deposits grow in size. Amyloid plaques mice are abundant in hippocampus and cortex of 1-year-old deltaE9 mice. At this age general axon degeneration and synapse loss are observed, along with impaired cognitive performance [18, 46]. Thus, loss of synapses coincides with decline in cognitive performance in these models.

Indeed, there is convincing evidence that not only the absolute spine number contributes to cognitive performance. In fact, dendritic spine size and shape are known to affect various functional parameters relevant for cognition, including spine motility, neurotransmitter receptor numbers and organelle abundance [33, 51]. Growing evidence shows that morphological changes of dendritic spines are associated with long-term synaptic plasticity (LTP) [68]. LTP increases spine head volume while shortening and widening spine neck [67]. This morphological plasticity allows generating changes in electrical properties of dendritic spines, which serve as isolated electrical compartments. For instance, it has been shown that shorter spine necks lead to larger depolarization while longer necks generate smaller somatic potentials [1]. It is believed that different types of memories need to obey different electrophysiological rules, and thus require morphological diversities of spines [51]. Along with changes in spine density, distinct alterations of spine morphology in APP23 mice and deltaE9 mice might also result in the different cognitive impairments described before [12, 18, 25, 31, 34, 45, 49, 60, 61]. Layer V pyramidal neurons in the somatosensory cortex are involved in motor learning [14, 64–66] and the formation of new dendritic spines correlates with the performance after learning [66]. While most behavioral tests focus on hippocampus-dependent memory tasks, the resulting behavior results from a complex interplay of various brain regions, in which somatosensory cortex neurons may play crucial roles. Thus, the alterations of dendritic spines which we found may well reflect part of the behavioral phenotype observed in these mice. Yet, the susceptibility of spines to the various toxic insults due to the overexpression of APP and its cleavage products may differ between brain regions, between different functional locations within a neuron (e.g. between apical and basal dendrites) or with the age of the experimental animals. Therefore, the relation of dendritic spine loss in layer V pyramidal neurons to cognitive dysfunction is not certain.

Compared to APP23 mice, deltaE9 mice harbor an additional transgene of a familial AD mutation in PS1 with a deletion of exon 9, accelerating the cleavage of APP and thereby Aβ formation. In consistence with previous studies [10, 16], extracellular amyloid plaques have developed in 4–5-month-old deltaE9 mice but not APP23 mice. Being the abnormal protein aggregates that characterize human AD, Aβ deposits are one of the biomarkers for AD neuropathologic assessment [26]. Aβ production and aggregation might initiate serial molecular cascades, thus lead to clinical AD [20]. This amyloid cascade hypothesis seems to be feasible in early-onset AD, which is known to be caused by mutations of genes that increase Aβ accumulation [27]. However, as early-onset AD only accounts for a few percent of AD cases and the correlation between cognitive decline and Aβ deposits is weak [2, 17], alternative explanations for the pathogenesis of AD have emerged [36, 40].

In contrast to age-matched deltaE9 mice, only a minor soluble Aβ burden was found in the brains of young APP23 mice [10, 37, 61]. Overexpressed APP in APP23 mice is predominantly localized intracellularly and the mechanisms of this aberrant accumulation and its relevance in sporadic AD need to be further investigated. Interestingly, a number of studies have reported increased amount of APP mRNA in AD patients [39, 41, 47], indicating that up-regulated transcriptional activity of APP may also contribute to AD pathophysiology. Moreover, accumulated APP has been found in dystrophic neuritis of AD [11, 54]. It is therefore tempting to speculate that intraneuronal accumulation of APP and/or its cleavage products including Aβ in AD may also contribute to synaptic damage [44, 58]. Indeed, an extra copy of the APP gene can cause neuronal dysfunction and symptoms similar to those seen in AD [42]. APP gene triplication in Down’s syndrome and APP locus duplication in rare families lead to clinical AD-like pathology in adults and result in early-onset dementia [21, 50]. The neurotoxicity of APP is largely thought to be caused by its proteolytic fragments. Besides Aβ, other proteolytic APP fragments, such as C83, C99 and APP intracellular domain, could also be involved in AD pathogenesis [55]. By regulating gene expression, these derivatives may give rise to neuronal degeneration [35, 55]. Additionally, through the direct interaction between APP and *N*-methyl-d-aspartate receptors (NMDARs), overexpressed APP up-regulates the expression of NMDARs and thus may contribute to neuronal toxicity by disrupting synaptic homeostasis [23].

To conclude, despite the fact that APP23 and deltaE9 mice show similar cognitive impairments and neuropathology in advanced age, our data clearly show different dendritic spine abnormalities in these two transgenic mouse models in young adulthood. Our findings imply that synaptic failure in these mouse models may be caused by different mechanisms in an age-dependent manner. Since the mechanisms underlying the development of sporadic AD are still uncertain, this study has significant implications for the analysis of distinct AD transgenic mouse models during preclinical drug evaluation for treatment of early-stage AD.

## Electronic supplementary material

Supplementary material 1 (EPS 1438 kb)

Supplementary material 2 (EPS 1214 kb)

Supplementary material 3 (EPS 6463 kb)

Supplementary material 4 (DOCX 18 kb)
