# Supplementary material for: Intraneuronal APP and extracellular Aβ independently cause dendritic spine pathology in transgenic mouse models of Alzheimer’s disease
Source: Acta Neuropathol. 2015 Apr 11;129(6):909–20. doi: 10.1007/s00401-015-1421-4 (PMC4436699; doi:10.1007/s00401-015-1421-4)
Supplement: Supplementary file 4 — Supplementary material 4 (DOCX 18 kb) [file 401_2015_1421_MOESM4_ESM.docx]

**Supplementary methods**

Western blot: Tricine-SDS-PAGE was used in western blot as described before [1]. Briefly, 10% cortical tissues (w/v) were homogenized in lysis buffer supplemented with protease inhibitors (Roche), followed by centrifugation at 500 rpm for 1 min. The supernatant was collected and protein concentrations were adjusted by Bradford assay (Sigma-Aldrich) to ensure the same amount of protein being loaded for each sample (100 µg). Samples were mixed with SDS-containing sample buffers and incubated at 37 °C for 20 min. After electrophoresis in 15% sample gel, proteins were transferred to a polyvinylidene difluoride membrane (Millipore). The APP/Aβ primary antibody, 6E10 (Convance), was used at 1:500 concentration for immunoblotting. Full-length APP and Aβ oligomers were determined based on the molecular weights [2]. Protein bands were quantified in ImageJ. Results were normalized to control and repeated measures one-way ANOVA was used followed by Newman-Keuls’s test.

**Supplementary figure legends**

Supplementary Figure 1. Dendrites at different distances from plaques in deltaE9 mice.

(a and b) In vivo overview images showing GFP-labeled dendrites (white) and methoxy-X04 labeled plaques (blue). Dendrites that were localized at plaque-free overview images are classified as deltaE9 (>100 µm, a) and the ones in close proximity to plaques are named as deltaE9 (<30 µm, b). Arrowheads point to the chosen dendrites for spine analysis.

Supplementary Figure 2. Young adult APP23 mice overexpress AP

(a and b) Western blot examples (a) and quantification of protein band (≈85 kDa, b) reveal an overexpression of APP in the cortex of APP23 mice.

(c, d and e) Quantifications of protein bands (≈23 kDa, ≈56 kDa and ≈115 kDa) reveal overexpressed Aβ in the cortex of deltaE9 mice, but not in APP23 mice. n=5 in each group. (b-e) *, p<0.05; **, p<0.01 (ANOVA with Dunnett’s post-hoc test).

Supplementary Figure 3. Increased intracellular APP accumulation is accompanied with decreased spine density and altered spine morphologies in the CA1 region of APP23 mice

(a) Maximum intensity projections of ex vivo images of GFP-labeled neurons (white, A and B) and intracellular APP accumulation in CA1 pyramidal neurons (black). Green dashed circle indicates the area of soma from GFP-labeled neurons. Arrow points at the chosen dendrites for spine analysis. Scale bar represents 20 μm.

(b) Maximum intensity projected basal and apical dendrites from A and B. Scale bar represents 5 μm.

(c, d and e) The dot plots are the intensity of intracellular APP in dendrites from CA1 pyramidal neurons versus spine density, mushroom and stubby fractions separately. Straight lines are fitted by nonlinear regression. Each dot represents one neuron. n=38. (c-e) **, p<0.01 (F test).

**References**

1 Schagger H (2006) Tricine-SDS-PAGE. Nature protocols 1: 16-22 Doi 10.1038/nprot.2006.4

2 Teich AF, Patel M, Arancio O (2013) A reliable way to detect endogenous murine beta-amyloid. PloS one 8: e55647 Doi 10.1371/journal.pone.0055647
